# The Normal 3D Gleno-humeral Relationship and Anatomy of the Glenoid Planes

**DOI:** 10.5334/jbsr.1346

**Published:** 2018-01-31

**Authors:** Tom Verstraeten, Lieven De Wilde, Jan Victor

**Affiliations:** 1 Department of Radiology, University Hospital Ghent, BE; 2 Department of Orthopaedic Surgery and Traumatology, University Hospital Ghent, BE

**Keywords:** 3D CT-scan, Shoulder arthroplasty, Anthropometric, Glenoid component positioning, Glenoid positioning device

## Abstract

Knowledge of the normal and pathological three-dimensional (3D) gleno-humeral relationship is imperative when planning and performing a total shoulder arthroplasty. Currently, two-dimensional (2D) parameters are used to describe this anatomy and despite the fact that these 2D measurements have a wide distribution in the normal population, they are commonly accepted. This broad distribution can be explained on one hand by anatomical factors and on the other hand, by positional errors. A 3D CT-scan reconstruction and evaluation can overcome this shortcoming and can be used to determine more accurately the surgical planes on the normal and pathological shoulder joint. There is, however, no consensus on which references should be used when studying this 3D relationship. This thesis describes the normal 3D gleno-humeral relationship and the best glenoid plane to use in surgery, based on 3D CT-scan. Furthermore, a glenoid aiming device that can be of surgical help in the reconstruction of the normal glenoid anatomy was developed based on these measurements.

## Introduction

The shoulder joint is a very complex joint because of its great mobility. Therefore, normal alignment of its structures is imperative for a good function as misalignment will result in malfunction. For example, with the knee joint, genu varum and genu valgum will result in faster degeneration of the cartilage of the knee joint with respectively medial and lateral gonarthrosis.

**Figure 1:**
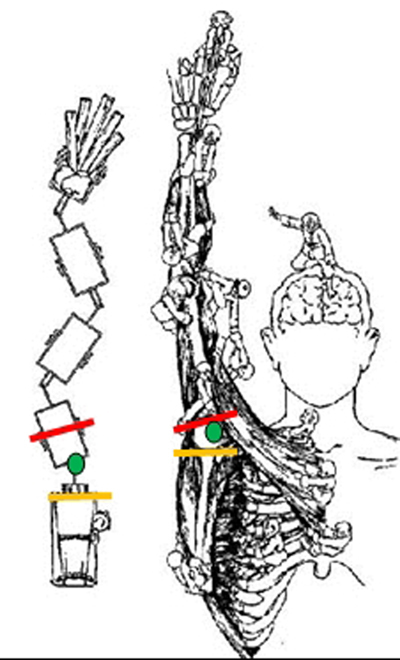
Shoulder function as described by Codman. The rear axis of the car represents the plane of the glenoid (yellow) and the axis of the trailer represents the plane of the humerus (red). The centre of rotation of the shoulder joint is the trailer hook in the figure (green).

So, in a normal gleno-humeral joint there is an optimal positioning of the bony humeral head and glenoid with an optimal balance between these bony structures and the muscles. We can compare this positioning with the figure of Codman (Figure 1) [1]. The rear axis of the car represents the plane of the glenoid and the axis of the trailer represents the plane of the humerus. The centre of rotation of the shoulder joint is the trailer hook in the figure. If the car wants to push back all the trailers in a correct manner, then all components need to be in the exact position to each other. This is the same for the shoulder joint. In complex movements like arm raising, all bony components need to be in a correct alignment to each other. The knowledge of this normal bony anatomy is therefore imperative to understand its function and to predict pathology. This has already been illustrated in previous studies [2] where a 10° malpositioning of the plane of the glenoid results in a 5mm translation of the humeral centre of rotation. In this thesis, we extensively described this bony anatomy.

### 2D parameters: Classical anatomical parameters

**Figure 2:**
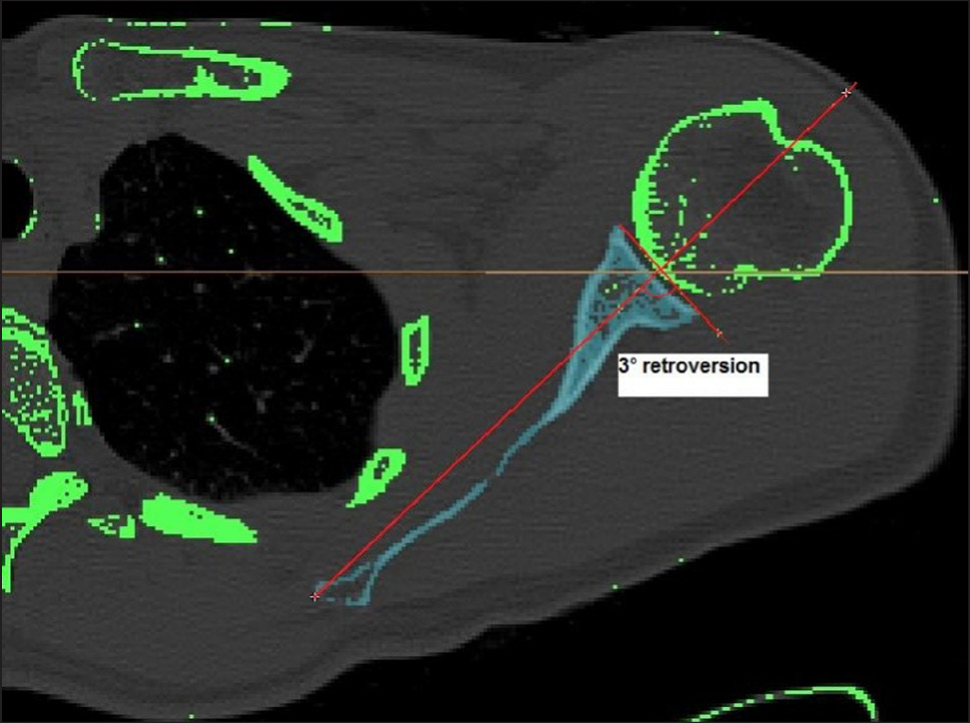
The glenoid version is the angulation of the glenoid to the transverse axis of the scapula in the axial plane of the body. In this example, the glenoid version measures 3° retroversion.

The classical parameters describing this bony anatomy, are two-dimensional (2D). They include the inclination and the version of the humerus and glenoid in respectively the coronal and axial planes [3,5] (Figure 2). Nevertheless, they have a wide distribution around an average value and seem therefore not too useful for surgery, a means which requires a greater accuracy.

This broad distribution can be explained by both anatomical factors and by positional errors [7,8].

One of the anatomical factors is the torsion of the glenoid from cranial tot caudal. This means that there is a relative retroversion of the glenoid when it is measured in the more cranial part of the glenoid than in the caudal part.

The positional factor means that the version of the glenoid can vary greatly with the position of the scapula on the CT-scan.

**Figure 3:**
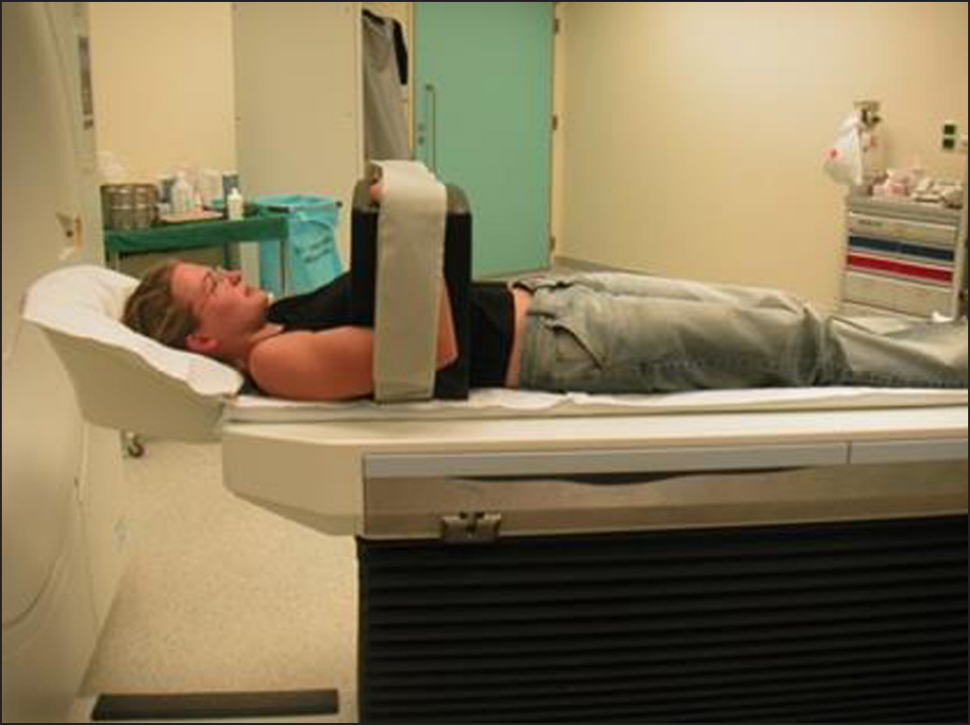
Position of the patient in CT-scan gantry. This position mimics the operative conditions and keeps the gleno-humeral joint in a neutral position.

A 3D CT-scan reconstruction and evaluation can overcome this shortcoming. When the measurements are performed between two bony structures, like in the gleno-humeral joint, the positional influence of the scapula can be minimalized by standardizing the patients positioning in the CT-scan gantry (Figure 3). This position mimics the operative conditions and keeps the gleno-humeral joint in a neutral position. In this thesis, all patients were scanned in this neutral position.

So these two factors (3D reconstruction images and the standardized position of the patient in the CT-scan gantry) can reduce the variability of the anatomical measurement and are already described in the literature.

### 3D parameters: planes of the humerus and the glenoid

**Figure 4:**
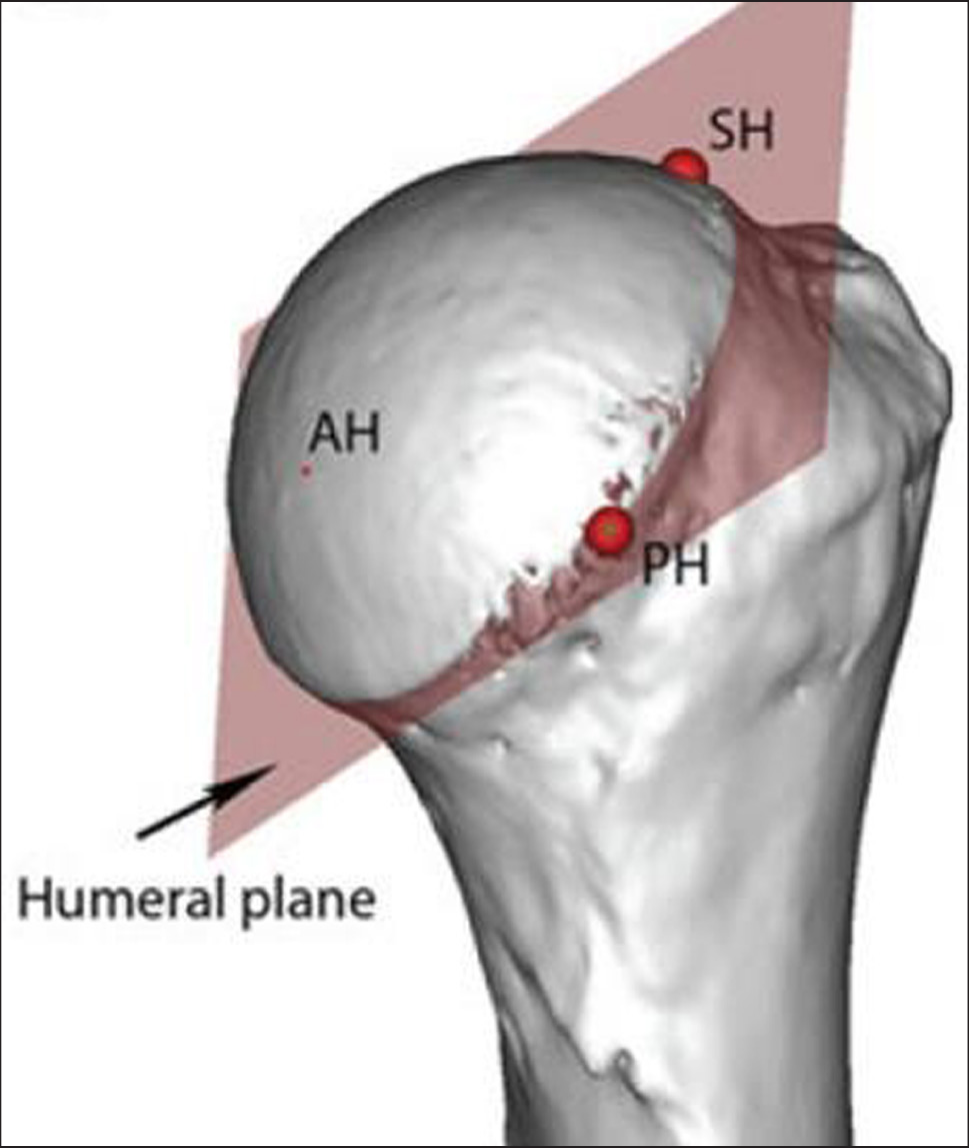
Humeral plane. This is a circular plane when a cut is made at the level of the anatomical neck of the humeral sphere.

3D anatomy enables improved geometrical fitting. The humeral sphere can be described comprehensively. A cut through this sphere at the level of the collum anatomicum defines a circular plane unavailable via 2D imaging. The introduction of the ‘native’ retroversion and inclination, guided by the anatomical neck of the humerus, improved the 3D restoration of the centre of rotation [4,6,9]. The explanation for this is that the use of the anatomical neck as new surgical reference (Figure 4) takes into account the native retroversion in combination with the native inclination. Moreover, using this humeral plane as the preferred surgical humeral landmark reduced the need for preoperative measurements because the anatomical neck is always identifiable on surgery, even in omarthrosis with severe destruction of the humeral head and osteophytes. Further, a firm relationship between the radius of the best-fitting circle of the anatomical neck and the head height helps in case of erosion and deformation of the humeral head to an improved individual restoration of the centre of rotation [4,6].

On the glenoid side, the classical parameters (angles between lines like retroversion and inclination), are still used because there is no consensus on which plane to restore. The fact that there is no consensus about which plane to use can be explained by the variability of the morphology of the glenoid. The glenoid has been described as comma-, pear- or teardrop-, round-, and ovoid-shaped [10]. Despite no reference plane having been described so far on the glenoid side, it is recognized that the inferior part of the glenoid constantly has the shape of a true circle [11,12].

### Centre of the glenoid plane

As we described the glenoid plane as the ‘rear axis’ of the car in the example of Codman [1] (Figure 1), we also need to define the centre of this plane as the position of the trailer hook on this rear axis. This centre of the glenoid plane is the centre of a certain geometrical plane shape. In the literature we find that ‘the centre of the glenoid fossa’ is not always well defined. What exactly is meant by ‘the centre of the glenoid’ since the morphology of the glenoid is not that of a geometrical shape?

The orthopedic surgeon tends to use the midpoint of the glenoid described as the crossing line between the most superior and inferior point of the glenoid and the largest antero-posterior distance. De Wilde et al. [11] described that only the peripheral rim of the inferior quadrants of the articular surface of the glenoid was found to be located on a circle. Furthermore, the definition of the centre of this circle appeared to be more reliable than determining the centre of the glenoid as the cross point of the cranio-caudal and antero-posterior axis of the glenoid as described by Saller [13]. Finding the 3D mathematical centre of the glenoid on 3D CT-scan reconstruction images seems to overcome the latter dilemma.

### Gleno-humeral relationship

Literature on the gleno-humeral relationship is scarce. De Wilde et al. [14] investigated the gleno-humeral relationship in a standardized reference system. To this purpose, it is necessary to establish valuable guidelines, based on the anatomic relationship of the gleno-humeral joint in the normal shoulder, to optimize the positioning of the prosthetic components in total shoulder arthroplasty. To create the intra-operative situation in a standardized fashion (Figure 2), the study subjects lay with their back flat on a hard surface, bringing the scapula in an individually reproducible position to the chest. A thoracobrachial orthosis was applied to position the arm adducted in the coronal plane and the forearm flexed in the sagittal plane of the body. They found a statistically significant correlation between the axis of the glenoid cavity and the axis of the humeral head. This correlation suggests that the orientation of the gleno-humeral joint is more constant, regardless of the orientation of, for example, the transepicondylar axis and the axis of the scapula (measurements between lines drawn in the same bones). The measurement of this correlation also approximates better a Gaussian distribution than the values of the version of the glenoid and the humeral head. Iannotti et al. [15] reported a strong linear correlation between the lateral humeral offset (distance from the base of the coracoid process to the most lateral point of the greater tuberosity) and the size of the humeral head. They concluded that different sizes of humeral prosthetic components are needed for the correct reconstruction of the lateral humeral offset to optimize the moment arm of the deltoid muscle and the rotator cuff.

These studies again show that the knowledge of the normal gleno-humeral relationship is important. They studied this relationship as correlations between lines at the humeral and at the glenoid side, which are 2D parameters. They did not study the gleno-humeral relationship in 3D.

So aside from these 2D findings of the gleno-humeral relationship, no studies exist to describe the gleno-humeral relationship in 3D. If this relationship would exist and be similar or less variable in 3D (the angle between the humeral plane and the glenoid plane), this information could be used for peroperative guidance of the glenoid plane based on the humeral plane.

## Discussion

In this thesis we only used bony reference points to describe the bony morphology of the glenoid and the gleno-humeral relationship. We are aware that the shoulder joint is a complex structure of not only bones but also ligaments and muscles to complete its function.

Nevertheless, the bony alignment that is based on the biomechanical behavior of the shoulder joint (ball with centre of rotation and a small glenoid circular plane), as described in this thesis fits perfectly with the comparison of a car with trailer driving in reverse mode as described in the introduction by Codman [1], were the glenoid plane is defined by the rear axis of the car and the centre of rotation with the trailer hook.

We know from the literature that the morphology of the glenoid and proximal humerus are very diverse. Several studies have already reported their normal configuration and anatomical characteristics. All of these studies measured angles between two lines, in the transversal plane (version) and in the coronal plane (inclination), which are 2D parameters. When we combine these two 2D parameters, we can define a plane shape (version × inclination). At the humeral side, this is called the collum anatomicum and is already been described in the literature [4,11]. It is this plane of the proximal humerus that the orthopedic surgeon uses to reconstruct the proximal humerus.

At the glenoid side, no such plane has been described. Therefore, the definition of the glenoid plane itself is not clear.

**Figure 5:**
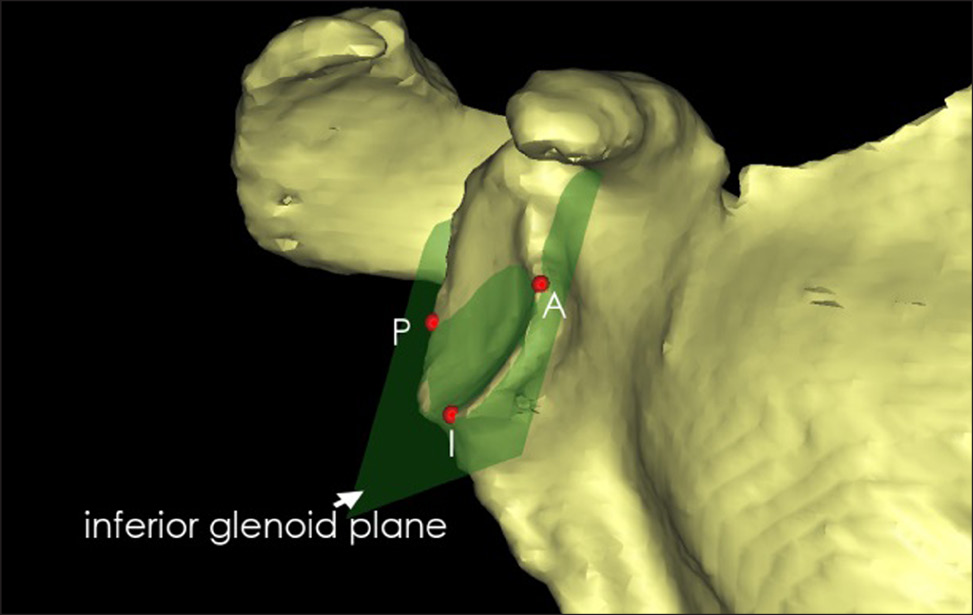
The inferior glenoid plane, created with an anterior (A), posterior (P) and inferior (I) point at the bony rim of the inferior two quadrants of the glenoid.

This is why the first aim of our thesis was to describe a plane shape on the glenoid side (this is the rear axis of the car in the example of Codman [1]). Literature suggests that, at the inferior glenoid plane, a constant shape of a true circle can be distinguished [11]. We wanted to investigate which glenoid plane (version × inclination), that can be defined by easily accessible bony landmarks, would provide the least variability in vivo and would be the most suitable for prosthetic surgery of the glenoid. Our results show that the inferior glenoid plane (created with the most anterior, posterior and inferior glenoid point at the bony rim of the inferior glenoid) as fulfilling these criteria versus all other planes (Figure 5). An explanation for this difference is probably the variable anatomy of the superior part of the glenoid with the tuberculum superius and the glenoid notch [10].

**Figure 6:**
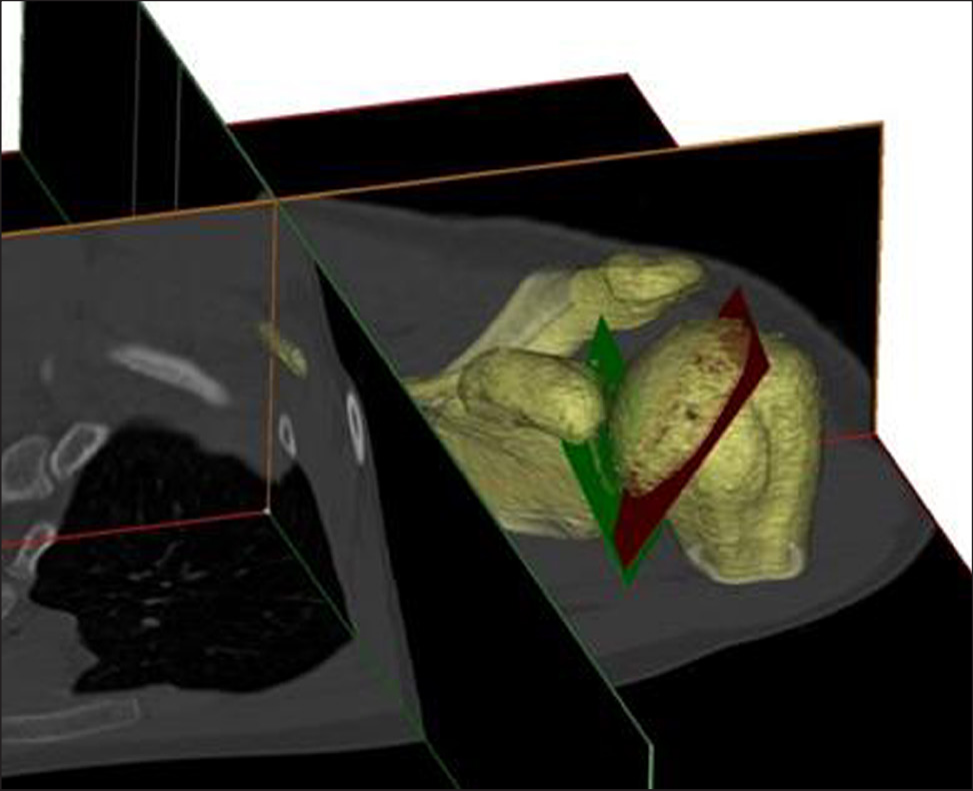
The gleno-humeral angle. This is the angle between the humeral plane (red plane) and the glenoid plane (green plane).

Our next aim was to describe a new 3D angle (Figure 6), which describes the 3D gleno-humeral relationship. This angle was defined as the angle between the humeral plane (which is the collum anatomicum already described in the literature [4]) and the glenoid plane of the individual patient. This glenoid plane was the inferior glenoid plane. This gleno-humeral angle had a mean value of 57.9° and a standard deviation of 6.71°. This variability was slightly smaller but not statistically different from the previously published 2D measurements [14], and the gleno-humeral angle has a Gaussian distribution. This new 3D anatomical information of the normal gleno-humeral relationship can be used to distinguish normal from pathological anatomy, as well as in alternative surgical guidance especially in bony-deficient glenoids. To our knowledge, this study was the first to determine the normal 3D gleno-humeral relationship between the humerus and the glenoid, described as the gleno-humeral angle. In this study, we standardized the position of the patient in the CT-scan to minimize the error of positioning. With these first studies we experienced difficulties to understand the so-called 3D retroversion/inclination angle. We could not exactly understand how this plane was orientated in the body space. A possible explanation for this is that, in the literature, the defined angles are described as the angle between the projection of these planes and are still 2D parameters [15]. So we concluded that we had to define in a more accurate way the difference between 2D and 3D parameters.

**Figure 7:**
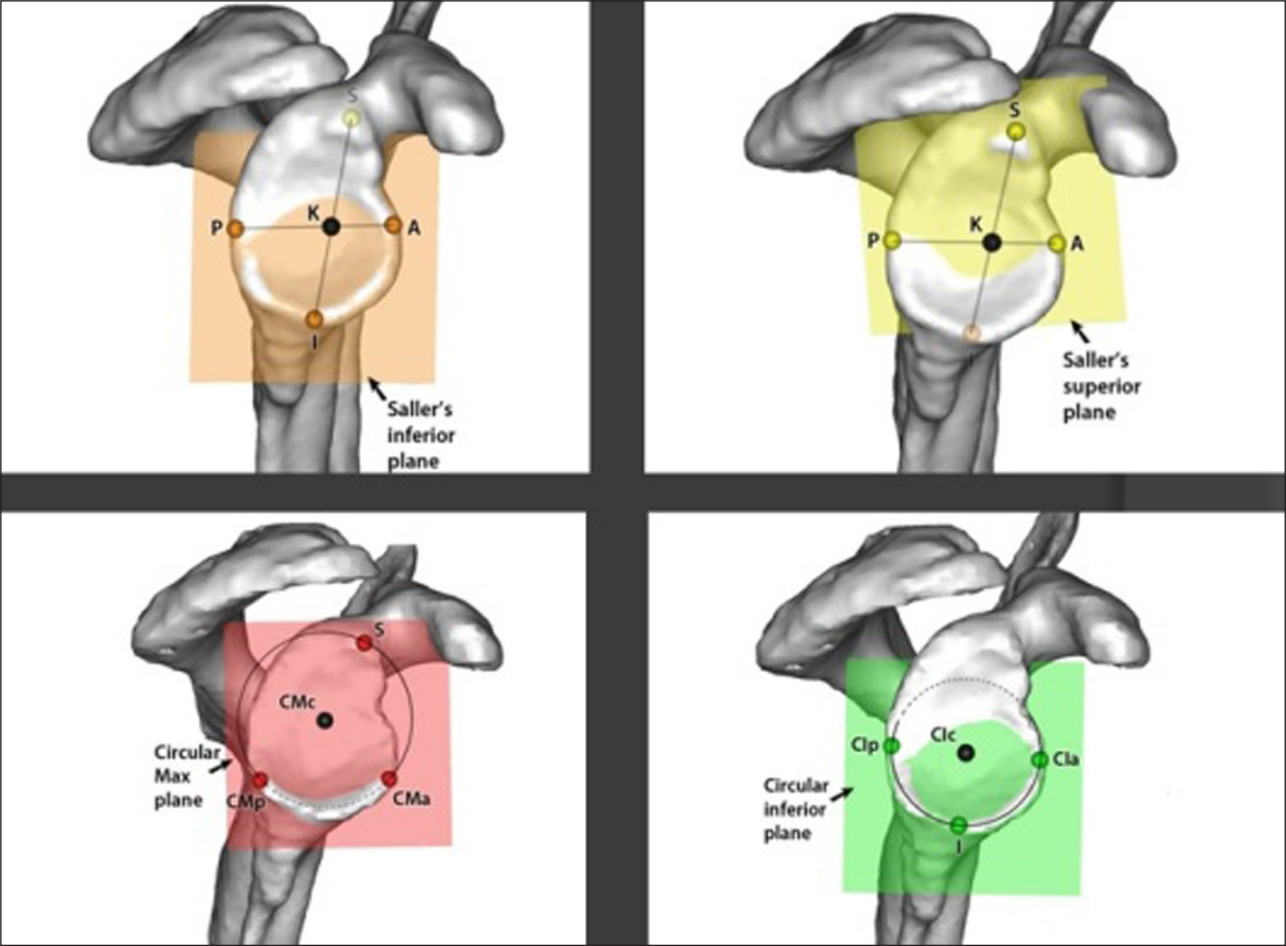
The centre of the glenoid was determined by two different methods. The first method defines the centre as the midpoint of the greatest antero-posterior en supero-inferior distance of the glenoid (top figures). With this method (according to Saller) we defined a triangular-shaped plane (Saller’s inferior and superior plane). For the second method we used circular planes. The circular planes are defined by the best-fitting circle constructed with three points. The Circular Max plane (bottom left) takes the whole glenoid into account and the Circular Inferior plane is located at the inferior two quadrants of the glenoid (bottom right).

To overcome this problem we introduced a Cartesian coordinate system to quantify the position of this plane. Such a 3D coordinate system needs a strict definition of a geometrical structure as well as the definition of its gravity centre. This centre can then be used as the midpoint of the coordinate system (this is the position of the trailer hook on the rear axis of the car in the example by Codman [1]). In this thesis, we compared triangular and circular shapes on the glenoid with a special attention to the inferior glenoid plane. These planes (two triangular planes and two circular planes) were each determined by three easily accessible bony surgical reference points and each plane had its own centre point. The position of this centre point was then evaluated in relation with the humeral centre of rotation. If we defined, as is classically done in the actual literature, the centre of the glenoid as the crossing point of a line between the most superior and inferior point of the glenoid and a line between the most anterior and posterior point of the glenoid (triangular planes) we measured great differences with the equilateral geometrical shapes (circular planes). So we found that a circular plane can accurately define the glenoid centre better than a triangular plane. Moreover, the circular inferior plane and its geometric centre seem to be the most reliable because of its overall better observer reliability and significantly lower variability (Figure 7). Because the literature [11] suggested the importance of the integrity of a circular plane at the inferior glenoid to assure an optimal gleno-humeral stability, it seemed logical to use the best-fitting circular plane formed by the rim of the inferior quadrants of the glenoid. In this way it became much easier to define and surgically identify a geometrical glenoid centre. So it was decided to use this circular shape and its midpoint as the basis of a Cartesian coordinate system. Doing so, we were able to define the midpoint of this circle as the origin and the Y-axis as the crossing line between this circular plane and the scapular plane. The X- and Z-axis are the perpendicular lines on the Y-axis. In this way it became possible to better understand the 3D gleno-humeral anatomy. Lewis and Armstrong [16] also described a method of evaluating the 3D glenoid orientation. They fitted a sphere to the glenoid face and its orientation was described by two angles analogous to version and inclination. They had similar results as our results with the glenoid inferior plane (average 3.2° retroversion with a SD of 3.4°). However, the use of a circular plane based on minimally three bony reference points on the (inferior) glenoid rim is directly applicable to the surgical setting, whereas the ‘best spherical fitting method’ is only suitable for CT-scan reconstruction. This method also doesn’t take the gleno-humeral relationship into account, whereas the method with the inferior glenoid plane measures the angles in relation with the centre of rotation of the humerus.

**Figure 8:**
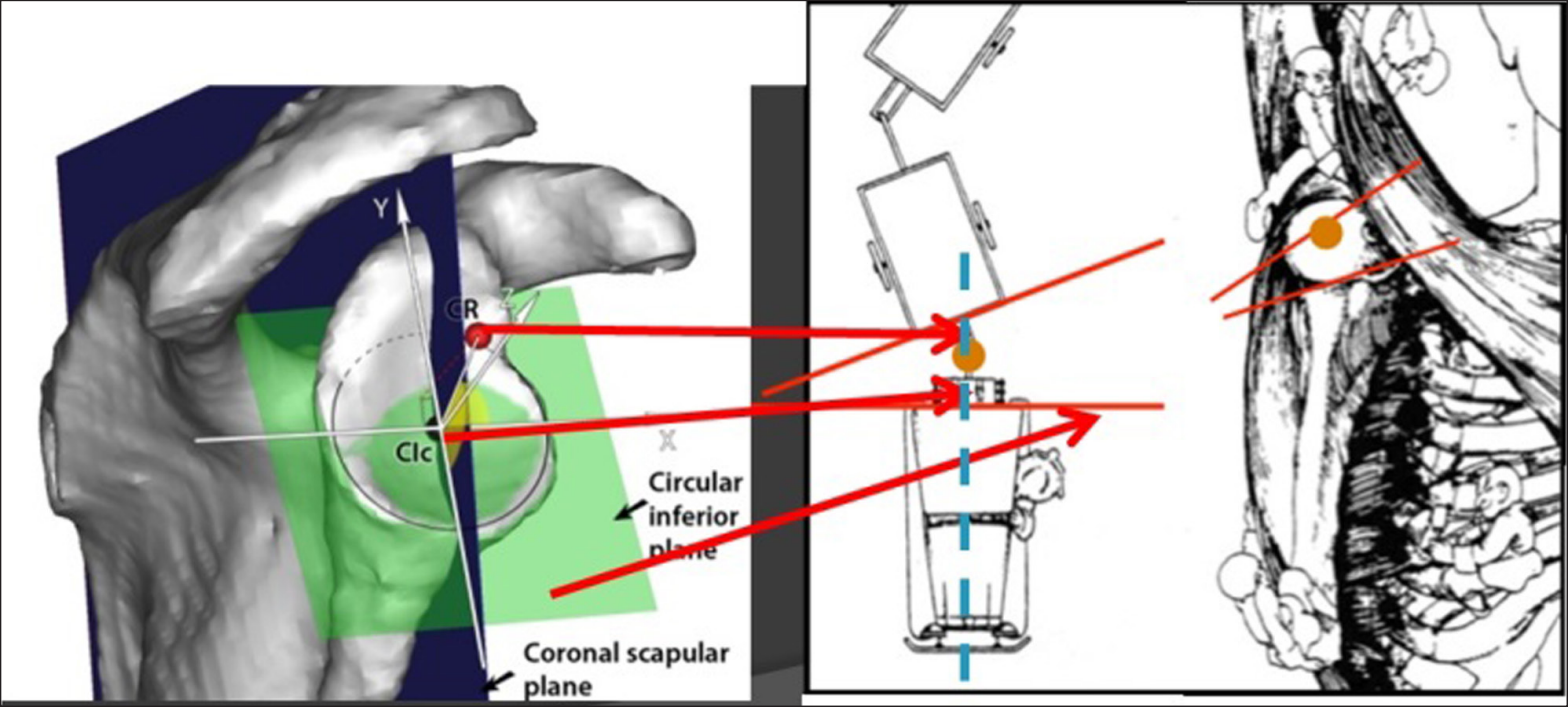
Position of the centre of rotation of the humerus (CR) to the centre of the inferior glenoid plane (CIc). The angle in the X-axis is 91.66° and in the Y-axis 91.7°. The distance is 24.8 mm. With this description, we quantified the position of the axis of the car and the trailer, and the position of the trailer hook with an angle and a distance.

So first we considered the bony anatomy on the humeral side of this glenoid plane. Doing so we were able to define the normal gleno-humeral relationship and quantify the centre of the glenoid to the centre of rotation. The angle of the centre of the glenoid to the cente of rotation of the proximal humerus is 91.66° in the X-axis and 91.7° in the Y-axis. The distance was 24.8 mm. With this description we quantified the position of the axis of the car and the trailer and the position of the trailer hook with an angle and a distance (Figure 8).

**Figure 9:**
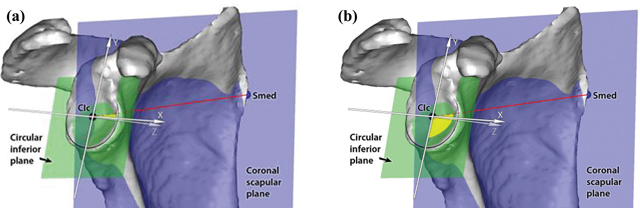
**(a, b)** Direction from the midpoint of the inferior glenoid circular plane to the most medial point of the scapula. The angle is 93.43° in the X-axis (left figure) and 111.36° in the Y-axis (right figure).

**Figure 10:**
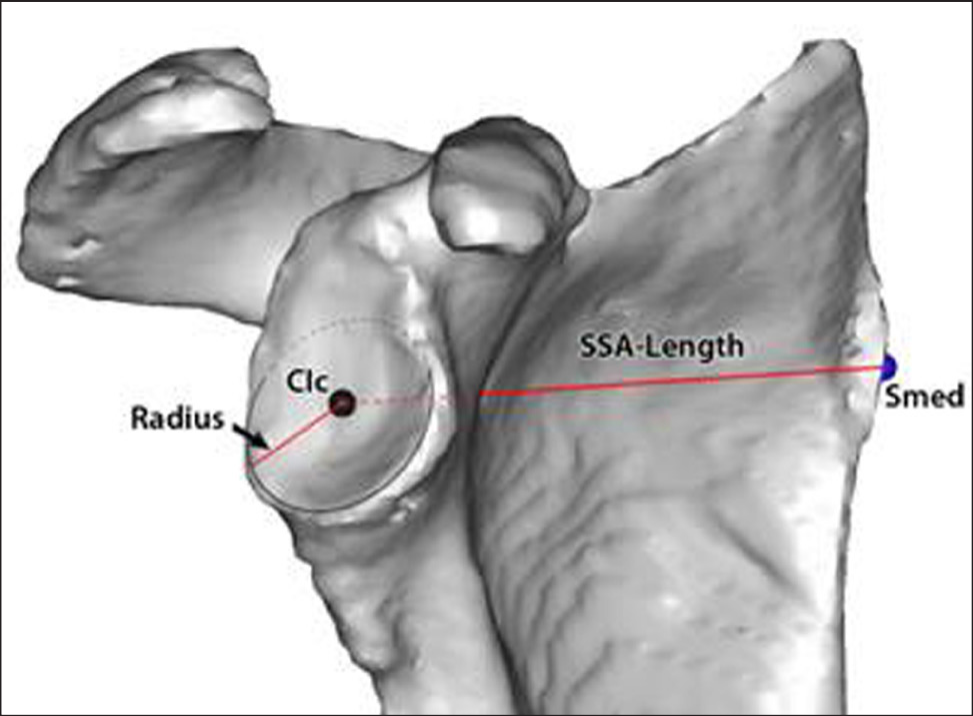
Correlation of the radius of the inferior circular plane with the spinal scapular length (r = 0.75).

Second, the bony anatomy at the scapular side of this glenoid plane was considered. Doing so, we were able to measure accurately the 3D orientation of the most medial point of the scapula. The angle of the centre of the glenoid to the medial point of the scapula is 93.43° in the X-axis and 111.36° in the Y-axis (Figure 9). Furthermore, a strong correlation was found between the radius of the best-fitting inferior glenoid circular plane and the length of the spina scapula (Figure 10). Similar to what is used and found at the humeral head, this finding can be useful in the reconstruction of the glenoid, where in more than 50% of the degenerative pathology, an important bony erosion can be found. So the direction from the midpoint of this inferior glenoid circular plane to the most medial point of the scapula in combination with the correlation of the radius of the inferior circular plane with the spinal scapular length can be helpful to define the native glenoid plane in case of erosion or destruction [17]. In comparison with the humeral side, we know from the literature that the 3D evaluation of the bony proximal humerus demonstrated that the collum anatomicum (or anatomic neck) is the best surgical reference for reconstruction of the proximal humerus [4,6]. This is, as mentioned earlier, the native humeral plane which combines the two 2D parameters of version and inclination. In case of erosion and deformation of the humeral head in pathology, a firm relationship between the radius of the best fitting circle of the collum anatomicum (after osteophyte removal) and the head height, can help in the restoration of the individual native humeral head.

This thesis also demonstrated that the inferior glenoid circle can be reconstructed with a minimum of three different points situated in a sector of 60° at the rim of the anterior part of the non-eroded part of the glenoid. This is only possible in posteriorly eroded glenoids. Shortcomings of this study are that this technique still needs a visual correction in order to prevent the native circle from exceeding the bony edges of the pathological glenoid. The knowledge of this study is also limited to shoulders where the glenoid erosion is restricted to less than a sector of 60° at the anterior part of the glenoid, which is not always the case in severe arthritic glenoids.

In the literature, several other techniques have been described to reconstruct the native glenoid plane. Lewis et al. [18] concluded that the internal shape of the normal glenoid vault (this is the space in the glenoid neck) has a uniform morphology. He described this shape in a geometric model and suggested five sizes that would fit an average clinical population. This 3D glenoid vault model was used as a template to predict normal glenoid version and to estimate the bone loss in gleno-humeral arthritis [18,19,20,21,22].

Ganapathi et al. [23] also described 3D CT-scan based measurements to predict native glenoid version. Therefore he used linear regression equations of the ‘Resch angle’, which is defined as the angle between the plane of the anterior glenoid wall and the plane of the glenoid fossa and the ‘anterior glenoid wall angle’, which is defined as the angle between the plane of the scapula and the anterior glenoid wall.

Most of these techniques to reconstruct the native glenoid are more complex than the technique described in this thesis. All other techniques need a careful preoperative 3D CT-scan reconstruction technique to define the native glenoid plane. The major advantage of the technique described in this thesis is that the surgeon can reconstruct this native glenoid plane by means of bony reference points which are easy accessible peroperatively and are situated in the visual field of the surgeon (three different points situated in a sector of 60° at the rim of the non-eroded anterior part of the glenoid). This results in a redundancy to use expensive software and to ease the complex preoperative planning. Because we were able, with our methodology, to accurately define the osteological 3D parameters, we were able to build a glenoid aiming device that can be of surgical help in the reconstruction of the normal glenoid anatomy as defined by our study of 150 normal shoulders.

**Figure 11:**
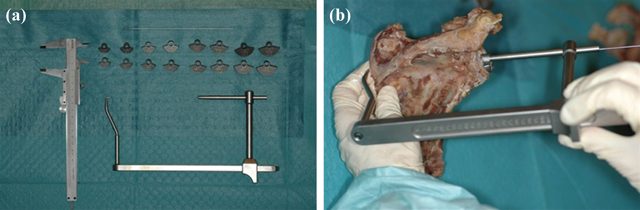
**(a, b)** Glenoid aiming device with its several components: K-wire, glenoid components, and the device.

**Figure 12:**
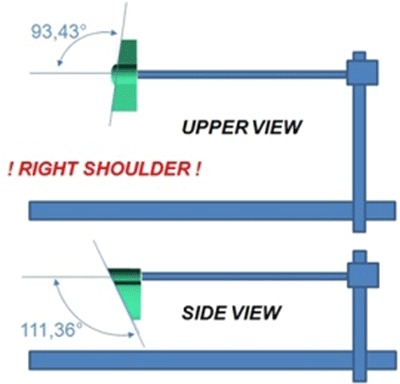
Fixed retroversion of the glenoid components of 3.4° and a fixed inclination of 111.36°.

**Figure 13:**
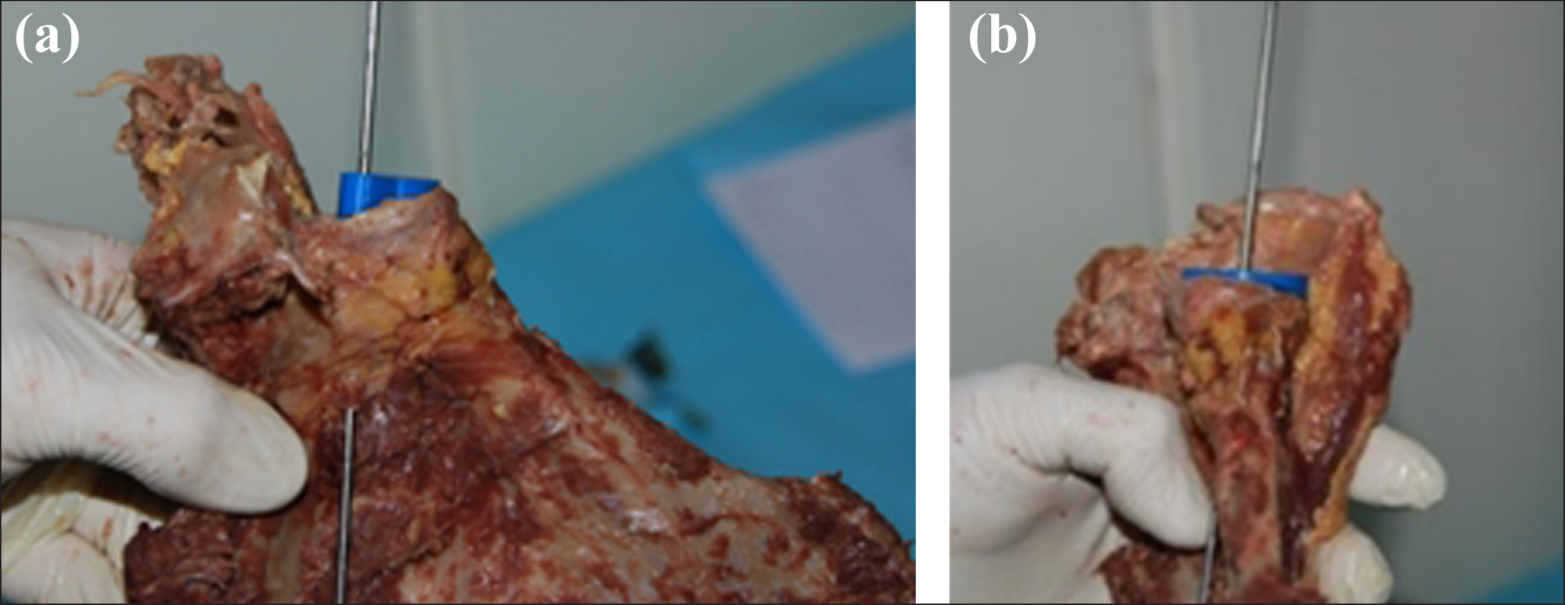
**(a, b)** Position of the glenoid component on the inferior part of the glenoid.

**Figure 14:**
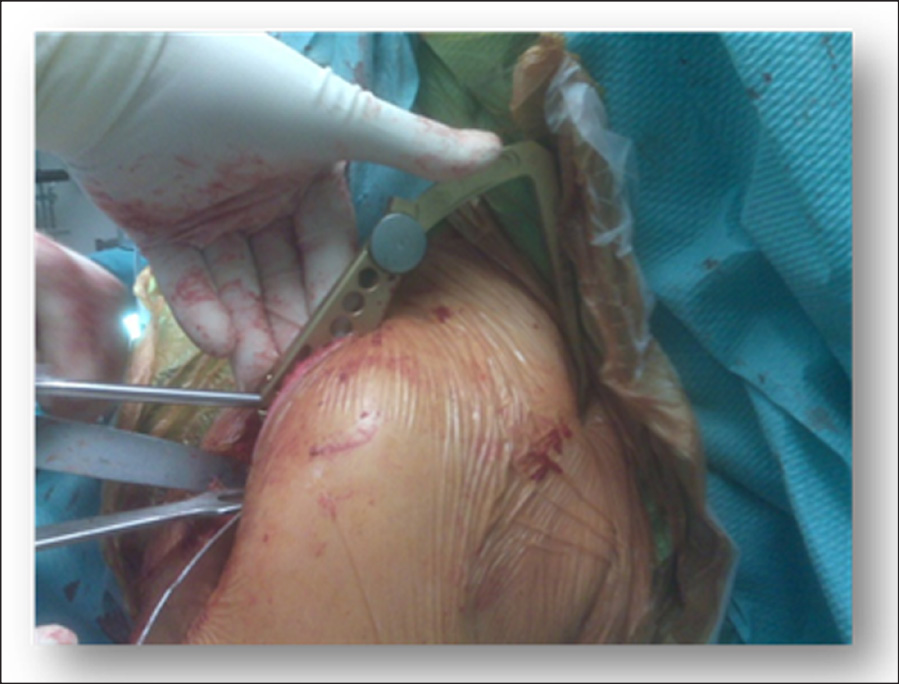
The use of the glenoid aiming device in practice.

We tested whether this extracorporeal aiming device could position a K-wire accurately in the center of the glenoid to provide guidance to ream the native glenoid plane. To guide the K-wire we used glenoid components that can be mounted onto the aiming device dependent on the size of the glenoid (11–17 mm) (Figure 11a and b). These glenoid components have a fixed retroversion of 3.4° and a fixed inclination of 111.36° based on the studies in this thesis (Figure 12). Therefore, in posteriorly eroded glenoids, the components can serve as a guide when they are positioned on the inferior glenoid to position the K-Wire. We created these glenoid components to fit the inferior glenoid circle because this part of the glenoid seems to have the least variability in 3D according to our previous studies (Figure 13). This guiding, together with the strong correlation which exists between the radius of the inferior circular plane and the spinal scapular length, can help the orthopedic surgeon to reconstruct the native glenoid plane. This extracorporeal guiding device is not patient specific but allows restoring the mean of the anatomy. Further study will decide whether this technique of extracorporeal guiding will be as efficient as the patient-specific [24] glenoid guidance in restoring the normal glenoid orientation (Figure 14).

## Conclusion

This thesis studied the 3D gleno-humeral relationship and the bony anatomy of the glenoid based on 3D CT-scan reconstruction images with the patient in a standardized position. This new 3D anatomical information can be used to distinguish normal from pathological anatomy, as well as in alternative surgical guidance to reconstruct the gleno-humeral joint.

We defined the inferior glenoid plane with its centre as the best surgical reference plane to use in surgery. Furthermore, a strong correlation was found between the radius of this inferior glenoid plane and the length of the spina scapula.

With this knowledge, we were able to build a glenoid aiming device that can be of surgical use in the reconstruction of the normal glenoid anatomy.
